# The Effects of EMMPRIN/CD147 on Late Function and Histopathological Lesions of the Renal Graft

**DOI:** 10.3390/biology11020232

**Published:** 2022-02-01

**Authors:** Magdalena Nalewajska, Martyna Opara-Bajerowicz, Krzysztof Safranow, Andrzej Pawlik, Kazimierz Ciechanowski, Sebastian Kwiatkowski, Ewa Kwiatkowska

**Affiliations:** 1 Department of Nephrology, Transplantology and Internal Medicine, Pomeranian Medical University, 70-111 Szczecin, Poland; nalewajska@gmail.com (M.N.); martyna.opara@wp.pl (M.O.-B.); kazcie@pum.edu.pl (K.C.); 2 Department of Biochemistry and Medical Chemistry, Pomeranian Medical University, 70-111 Szczecin, Poland; krzysztof.safranow@pum.edu.pl; 3 Department of Physiology, Pomeranian Medical University, 70-111 Szczecin, Poland; pawand@poczta.onet.pl; 4 Department of Obstetrics and Gynecology, Pomeranian Medical University, 70-111 Szczecin, Poland; sebastian.kwiatkowski@pum.edu.pl

**Keywords:** EMMPRIN, CD147, renal transplantation, renal function, fibrosis

## Abstract

**Simple Summary:**

This study provided innovatory data regarding the role of EMMPRIN in long-term renal graft function and renal biopsy specimens in the form of interstitial fibrosis/tubular atrophy. The main cause of renal fibrosis is identified to be the activation and accumulation of fibroblasts and myofibroblasts in the interstitium, surrounded by increased amounts of extracellular matrix, and EMMPRIN has been proposed as a contributor factor. The study has evidenced that EMMPRIN displays adverse effects on renal graft survival in terms of the frequent occurrence of DGF, poorer short-term and long-term renal graft function, more profound fibrotic lesions in biopsy specimens, and the degree of proteinuria. This represents an opportunity for more accurate prediction of the post-transplant period and early, non-invasive detection of kidney graft dysfunction. Future studies need to further investigate the clinical significance of the presented results.

**Abstract:**

Chronic kidney disease (CKD) is associated with renal fibrosis, and develops with the participation of fibroblasts and myofibroblasts from epithelial-to-mesenchymal transition (EMT). In cancer research, the key role of the glycoprotein CD147/EMMPRIN (extracellular matrix metalloproteinase inducer) in EMT has been proven. In this study, we evaluate how serum CD147/EMMPRIN affects long-term renal graft function and renal biopsy specimen lesions. In total, 49 renal graft recipients who had a renal biopsy within the last 18 months were retrospectively reviewed. At their most recent appointments, their serum concentrations of CD147/EMMPRIN and renal function were assessed. The occurrence of delayed graft function (DGF), estimated glomerular filtration rate (eGFR) at 1-year post-kidney transplantation (Tx) and the subsequent years of the follow-up period, and renal biopsy specimen lesions, mainly those related to renal fibrosis and tubular atrophy, were also evaluated. Results: CD147/EMMPRIN serum concentration correlated negatively with eGFR at the most recent appointment (ME 69 months) and with eGFR at 1 and 2 years after Tx (*p* < 0.05, R = −0.69, R = −0.39, and R = −0.40, respectively). CD147/EMMPRIN serum levels correlated positively with urine protein concentrations (*p* < 0.05, R = 0.73). A positive correlation was further found with the severity of renal biopsy specimen lesions such as interstitial fibrosis (CI), tubular atrophy (CT), double contours of the GBM (CG), mesangial matrix expansion (MM), and arteriolar hyalinosis (AH) (*p* < 0.05, R = 0.39, R = 0.29, R = 0.41, R = 0.32 and R = 0.40, respectively). Patients with a history of DGF had higher CD147/EMMPRIN serum concentrations (<0.05). Conclusions: CD147/EMMPRIN is linked to poorer long-term renal graft function. Additionally, a high serum concentration of CD147/EMMPRIN affects interstitial fibrosis tubular atrophy (IF/TA) lesions and proteinuria.

## 1. Introduction

Despite different clinical causes of chronic kidney disease, each case is associated with the damaging effects of fibrosis [1]. Additionally, loss of renal graft function is linked to its fibrosis, followed by secondary tubular atrophy (TA). The main cause of renal fibrosis is identified to be the activation and accumulation of fibroblasts and myofibroblasts in the interstitium, where they are surrounded by increased amounts of extracellular matrix (ECM). Renal myofibroblasts emerge de novo during renal fibrosis and represent the phenotype of fibroblasts that result from differentiation caused by cellular stress [2]. This type of fibroblasts or myofibroblasts come from the so-called residential quiescent tissue fibroblasts and vascular pericytes or originate in epithelial-to-mesenchymal transition (EMT) and bone marrow [3]. In EMT, epithelial cells transform into collagen-producing mesenchymal stem cells. The role of EMT in chronic kidney disease (CKD) progression is commonly recognized [4,5]. Many reports on cancers, their infiltration, and the development of metastasis caused by EMT have attributed a considerable role to a glycoprotein known as cluster of differentiation 147 (CD147) or extracellular matrix metalloproteinase inducer (EMMPRIN). It is acknowledged as a marker for acute kidney injury (AKI). However, little is known about its role in chronic kidney injury, while there are almost no reports on its activity in the renal graft. Only Kemmner et al. [6] have reported the association between EMMPRIN expression and chronic histopathological changes in renal grafts. This paper evaluates how the serum concentration of CD147/EMMPRIN affects long-term renal graft function and renal biopsy specimen lesions related to its chronic injury in the form of interstitial fibrosis/tubular atrophy (IF/TA).

## 2. Patients and Methods

### 2.1. Patients

In total, 49 renal graft recipients who were in the care of the Clinical Department of Nephrology, Transplantology and Internal Medicine, Pomeranian Medical University, Szczecin, Poland were retrospectively reviewed. The study included patients who reported within two weeks in 2019 for a standard follow-up appointment. As inclusion criteria, the patients were expected to have received their kidney transplant at least 1 year before and to have had a renal biopsy specimen taken within the last 12 to 18 months. The patients were after renal transplantation for an average of 69 months (ME, median). After kidney transplantation (Tx), all the patients received triple immunosuppressive therapy with glucocorticosteroids, a calcineurin inhibitor—tacrolimus or cyclosporin, and mycophenolate mofetil. In some of the patients, the steroid therapy was discontinued during the long-term follow-up. The parameters that were tested were the highest value of eGFR (estimated glomerular filtration rate, ZENITH GFR) achieved within the first 6 months post-Tx, creatinine concentration and eGFR at the most recent appointment, and creatinine concentration and eGFR at 1, 2, 3-, 4-, 5- and 10-years post-Tx. The GFR was estimated using the MDRD formula, as it has been reported to be the most suitable formula for GFR estimation in the late stages of CKD [7,8,9]. Moreover, the occurrence of delayed graft function (DGF), defined as the need for hemodialysis within the first week after transplantation, was evaluated. Urinalysis was carried out with a focus on the presence of urine protein (mg/dL). At the most recent appointment, the plasma concentration of CD147/EMMPRIN was also evaluated. The renal biopsy specimens were examined for such irregularities as interstitial inflammation (I), tubulitis (T), glomerulitis (G), peritubular capillaritis (PTC), the presence of C4D, interstitial fibrosis (CI), tubular atrophy (CT), vascular fibrous intimal thickening (CV), double contours of the GBM (CG), mesangial matrix expansion (MM), arteriolar hyalinosis (AH), and hyaline arteriolar thickening (AAH). The degree of changes in biopsy specimens was assessed according to a 2018 Reference Guide to the Banff Classification of Renal Graft Pathology. The thresholds for evaluated Banff Lesions Score have been presented in Table 1. Such recipient characteristics as age, sex, time from Tx, the body mass index (BMI) at the time of Tx and the most recent appointment, PRA (panel reactive antibody), CIT (cold ischemia time), and the numbers of mismatches for different types of HLAs (human leukocyte antigen) were evaluated. Patient details are presented in Table 2. The detailed characteristics of the recipients in the groups with and without chronic changes in the biopsy specimens have been presented in Table 3. Chronic changes in biopsy specimens have been defined as CI ≥ 2 and/or CT ≥ 2. Short-term renal graft function was defined as eGFR at 1- and 2-years after Tx, and long-term as eGFR assessed at last appointment—69 months (5.75 years) post-Tx at average.

### 2.2. Methods

Plasma samples were centrifuged at 4000 rpm and the time between blood sampling and centrifuging did not exceed 10 min. Sediment-free plasma was stored at a temperature of −80 °C awaiting analysis no longer than 14 days. EMMPRIN plasma concentration was assessed according to the manufacturer’s instructions using customized magnetic bead-based multiplex Luminex screening immunoassay kits (R&D Systems). MFI values ranged from 10–20,000, which represents the concentrations of evaluated analyte between 30–30,000 pg/mL. Values below or above the standard curve were to be rejected. All subjects gave their informed consent for inclusion before they participated in the study. The study was conducted following the Declaration of Helsinki and the Declaration of Istanbul and the protocol was approved by the Ethics Committee of Pomeranian Medical University, Szczecin, Poland approved the study protocol—KB-0012/23/18 (05FEB2018).

### 2.3. Statistical Analysis

We used Statistica 11 software (StatSoft, Tulsa, OK, USA) for statistical analysis. The Shapiro–Wilk test was used to study the distribution. The distribution of EMMPRIN was significantly different from normal (*p* < 0.05). We used a nonparametric Mann–Whitney U test to compare the two groups. Spearman’s rank correlation test was used to study correlations. Data that were not normally distributed were shown as the median [minimum–maximum]. *p*-values were significant if <0.05. 

## 3. Results

**Figure 1:**
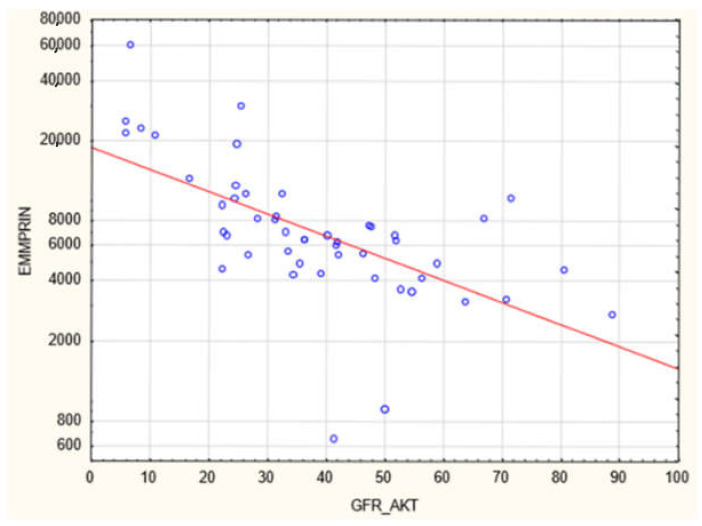
Negative correlation (R = −0.69, *p* < 0.05) between EMMPRIN/CD147 concentration and eGFR at the most recent appointment (ME 69 months).

**Figure 2:**
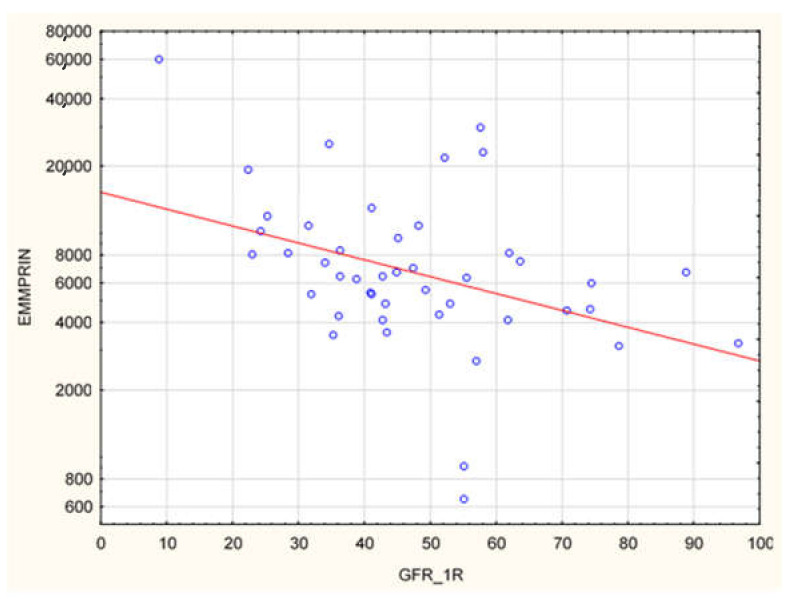
Negative correlation (R = −0.39, *p* < 0.05) between EMMPRIN/CD147 serum concentration and eGFR at 1 year after renal transplantation.

**Figure 3:**
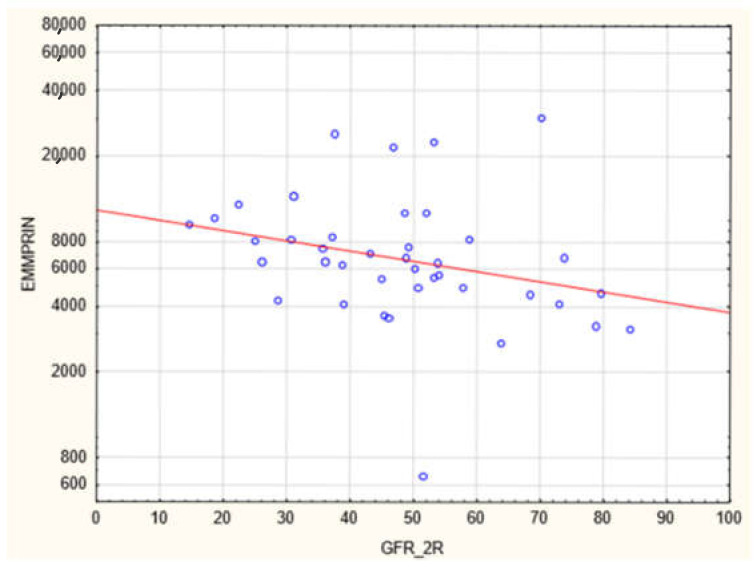
Negative correlation between (R = −0.40, *p* < 0.05) EMMPRIN/CD147 serum concentration and eGFR at 2 years after renal transplantation.

**Figure 4:**
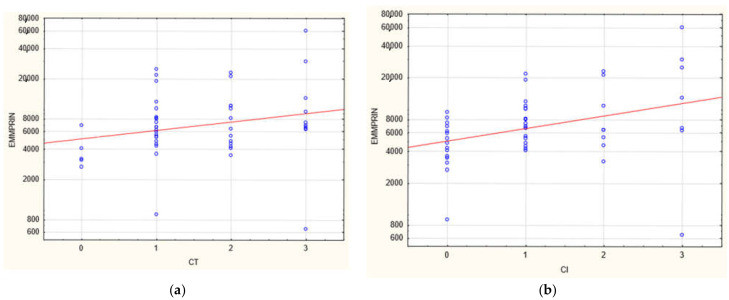
Positive correlation between EMMPRIN/CD147 serum concentration and severity of biopsy specimen lesions such as CI (**b**) and CT (**a**) (R = 0.39 and 0.029, respectively, *p* < 0.05).

**Figure 5:**
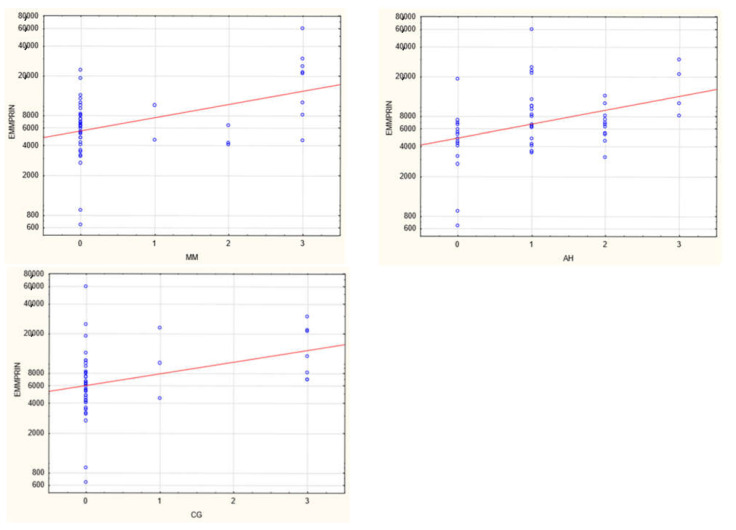
Positive correlation between EMMPRIN/CD147 serum concentration and severity of biopsy specimen lesions such as CG, MM, and AH (R = 0.41, R = 0.32, R = 0.40, respectively, *p* < 0.05).

The plasma concentration of CD147/EMMPRIN correlated negatively with eGFR at the most recent appointment, at 1- and 2-years post-Tx (*p* < 0.05, R = −0.69, R = −0.39, R = −0.40, respectively), as shown in Figure 1, Figure 2 and Figure 3. CD147/EMMPRIN concentration correlated positively with urine protein levels (*p* < 0.05, R = 0.73). A positive correlation with the exacerbation of renal biopsy specimen lesions, such as CI, CT, CG, MM, and AH, was found (*p* < 0.05, R = 0.39, R = 0.29, R = 0.41, R = 0.32, R = 0.40, respectively), as shown in Figure 4 and Figure 5.

The study group was divided into one group of patients with a history of DGF and another one of those with immediate renal graft function. It was established that the DGF group had significantly higher EMMPRIN serum levels (*p* < 0.05), as shown in Table 4.

## 4. Discussion

### 4.1. EMMPRIN/CD147

EMMPRIN, currently referred to as CD147 or EMMPRIN/CD147, is a membrane glycoprotein, which was originally discovered in various animal tissues and thus received different names. The name EMMPRIN was given to its human equivalent, and it has been kept in use to underline its significant role in regulating metalloproteinases. It has been proven to stimulate MMP-1 (Matrix metalloproteinase-1), MMP-2 (Matrix metalloproteinase-2), and MMP-3 (Matrix metalloproteinase-3). EMMPRIN has been postulated to be involved in ECM remodeling in both physiological processes and diseases [10,11]. Its role in AKI secondary to ischemia and CKD progression has been observed. It is found in the kidney on the basolateral side of tubular epithelial cells [12].

### 4.2. CD147/AKI

Ischemia-reperfusion injury (IRI) of the renal allograft is the main cause of DGF. In an experimental study, IRI induced in murine models deprived of the gene coding for CD147 led to a considerable reduction in the infiltration by neutrophils and macrophages and, consequently, a substantial drop in the degree of tubulointerstitial injury than in the models carrying that gene [12]. This experiment proved EMMPRIN’s key role in exacerbating the inflammatory infiltration in this type of injury. By pharmacologically inhibiting CD147, Seizer prevented infiltration by neutrophils and macrophages/monocytes following myocardial infarction [13]. Its proinflammatory activity may be associated with some interaction with cyclophilin A (a ligand of CD147), which plays a crucial role in regulating inflammation. This mechanism has been proven to take place in numerous in vivo experiments in sepsis-induced AKI, bronchial asthma, lipopolysaccharide-induced lung injury, and collagen-induced arthritis [14]. There are no reports on CD147 in the context of renal graft IRI, but its role in spreading inflammation can be deduced from the native kidney IRI model. Elevated CD147 concentrations are present in AKI [15]. Moreover, its concentration has been observed to positively correlate with creatinine levels. Serum CD147 originates in soluble CD147 from leukocytes, and in urine, CD147 is related to a tubular injury. Based on clinical research, this compound is believed to be the first candidate for a biomarker allowing the diagnosis of AKI [12,15]. The occurrence of DGF is associated with a 2.9-times higher risk of renal allograft failure in the long-term follow-up, hence its importance as a prognostic factor. Additionally, some authors have reported that DGF increases the incidence of acute kidney rejection episodes [16]. In this study, patients with DGF during the early post-Tx period had far higher CD147/EMMPRIN serum concentrations in the late post-Tx period. This phenomenon could be associated with the activation of CD147 and its maintained increased expression in the kidney transplant in the long-term follow-up. In acute renal reperfusion injury, fibrotic processes due to EMT begin in the kidney, where CD147 plays a major role.

### 4.3. CKD/EMT

Although there are various clinical causes of CKD, progression in each case is associated with the damaging effects of fibrosis [1]. Renal fibrosis is characterized by tubulointerstitial fibrosis, glomerulosclerosis, and destruction of the renal structure. The main cause of renal fibrosis is found to be the activation and interstitial accumulation of fibroblasts and myofibroblasts surrounded by excessive amounts of ECM. Renal myofibroblasts emerge de novo during renal fibrosis and represent the phenotype of fibroblasts that result from differentiation caused by cellular stress [2]. This type of fibroblasts or myofibroblasts come from the so-called residential quiescent tissue fibroblasts and vascular pericytes or originate EMT and in bone marrow [3]. In EMT, epithelial cells transform into mesenchymal stem cells. They acquire the capability to produce collagen. For EMT to develop fully, the basement membrane to which the epithelial cells adhere must be injured and the cells must migrate to the interstitium. EMT is the fundamental mechanism by which the kidney becomes fibrotic during the CKD process. It is also present in such physiological situations as embryogenesis. In pathological states, its main role consists in causing organ (mostly lung) fibrosis in the course of inflammation and initiating cancer infiltration and metastasis [17]. The sort of EMT related to inflammation is a type of wound healing and scar formation and is referred to as type 2 EMT. If the inflammation induced by injury persists, fibroblast numbers are up dramatically and the organ’s structure is damaged, which state is referred to as chronic type 2 EMT [17]. As mentioned above, basement membrane damage is one of the main stages of EMT, as it allows the transformed cells to migrate to the interstitium. MMP-2 is the key compound responsible for this phenomenon [17]. Since EMT was first discovered by Elizabeth Hay in 1960, many authors have proven its leading role in renal fibrosis and destruction in animal and human biopsy specimens [17].

### 4.4. EMT/CD147/EMMPRIN

**Figure 6:**
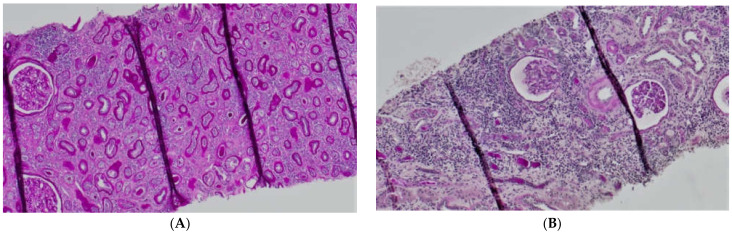
Histopathological images showing chronic changes graded as ci3, ct3 (**A**,**B**).

**Figure 7:**
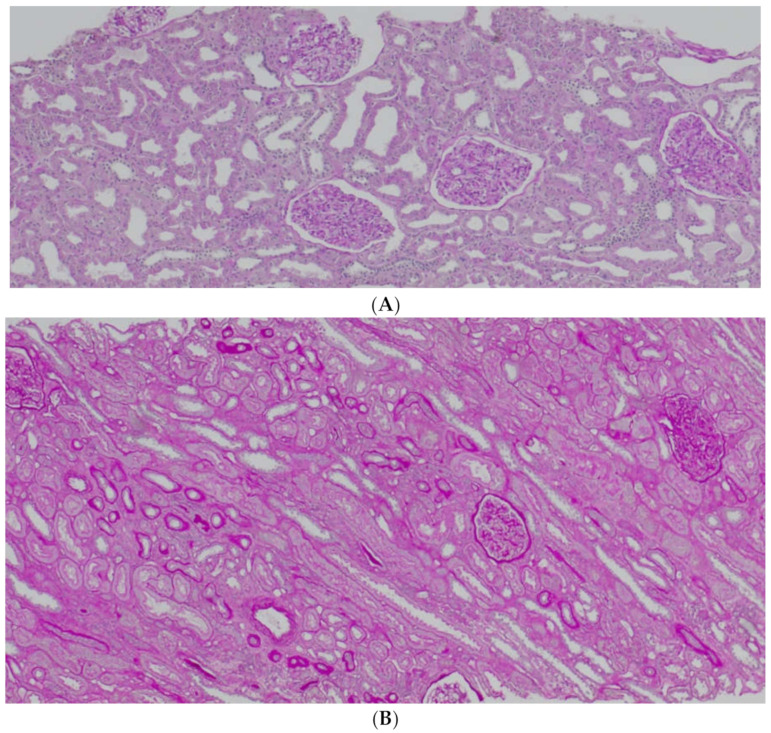
Histopathological images showing chronic changes graded as ci0, ct0 (**A**,**B**).

**Figure 8:**
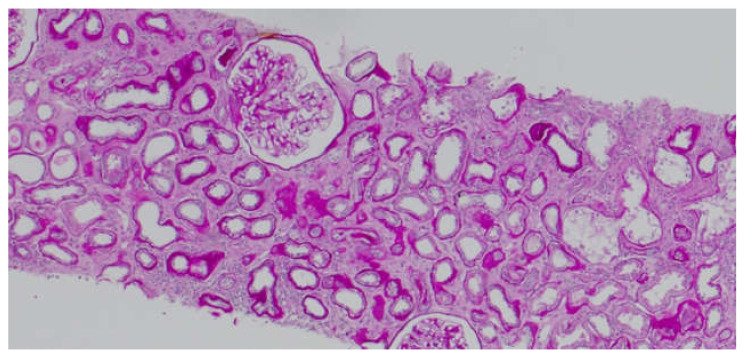
Histopathological image showing chronic changes graded as ci3, ct1.

It is known that CD147 abundant in the renal tubules is a very strong activator of MMP-2—the basement membrane-degrading metalloproteinase required for EMT to take place [6]. An in vitro experiment showed that in renal tubular epithelial cells CD147, together with MMP, induces the production of hyaluronic acid, which participates in the differentiation of the tubular epithelial cells into myofibroblasts in response to TGF-β (transforming growth factor β). Hyaluronan promotes the emergence of proteins typical of fibroblasts on the surface of the transforming cells [18]. Similar to AKI, CD147’s role in stimulating inflammatory infiltration by macrophages and monocytes in chronic kidney injury has been proven [19]. CD147 is thought to exacerbate fibrosis following three mechanisms: hyaluronan induction, MMP (particularly MMP-2) induction, and inflammatory infiltration. Our study showed that the concentration of CD147/EMMPRIN correlated negatively with eGFR at the time of sampling—69 months post-Tx on average. The level of serum CD147/EMMPRIN at the end of the follow-up period correlated negatively with eGFR at 1- and 2-years post-Tx, as well. These results prove that its high activity is most apparently linked to worse renal graft function since the very beginning—the early post-transplantation period and at 1- and 2-years post-Tx. Kemmner et al. [6] came to similar conclusions after they analyzed the presence of CD147 in biopsy specimens collected from renal graft recipients (50 months post-Tx on average) in the context of renal graft function and chronic histopathological lesions in the form of IF/TA. They found that abundant CD147 in the biopsy specimen was associated with more pronounced FT/TA lesions, poorer renal graft function evaluated using eGFR values, and an unfavorable change of renal graft function over time [6]. Another researcher, Yoshiko Mori et al., monitored the correlation of urine and plasma CD147 concentrations with renal graft function in patients with different kidney diseases [20]. There is very little information available on EMMPRIN expression in the renal tissue, especially in renal grafts. Most research into this compound is related to cancer and cancer invasion [21]. In many cancers, the effect of EMMPRIN on EMT processes linked to cancer invasion has been proven [21]. EMMPRIN plays a proven role in lung fibrosis secondary to inflammatory diseases [22]. Similar to Kemmner’s results, in our study EMMPRIN correlated positively with IF/TA histopathological lesions. CI demonstrated four degrees of lesion severity (0: ≤5%, 1: 6–25%, 2: 26–50%, 3: >50%), similarly to CT (0: no atrophy, 1: ≤25%, 2: 26–50%, 3: >50%). The more severe interstitial fibrosis and tubular atrophy, the higher the CD147 concentration. Histopathological images of kidney biopsy specimens of two patients with advanced chronic changes in terms of IF/TA have been presented below. In both biopsy specimens, changes have been graded as ci3, ct3, and EMMPRIN concentrations were 12,826 pg/mL and 29,709.47 pg/mL, respectively (Figure 6A,B). Other histopathological images present kidney grafts with no chronic changes. Ci and Ct grades were 0 with less than 5% of the kidney parenchyma being covered with fibrotic changes, and less than 5% of the tubules being atrophic. EMMPRIN concentrations were 3206.734 pg/mL and 897.41 pg/mL, respectively (Figure 7A,B). Another image presents advanced fibrosis (Ci3) with >50% of the kidney parenchyma covered, with <25% of the tubules being atrophic (Ct1). EMMPRIN concentration was 7304 pg/mL in this case (Figure 8).

Corresponding results were obtained by Shiren Sun et al., who studied the severity of IF/TA lesions in renal biopsy specimens collected from IgA nephropathy patients and found that an increased expression of CD147 was associated with more severe chronic lesions. Surprisingly, in our study, kidney allograft function did not significantly differ between groups with and without chronic changes defined as CI ≥ 2 and/or CT ≥ 2. The possible explanation is that the degree of fibrosis and the atrophy of the tubules in a kidney biopsy do not perfectly reflect on the histopathological image of the entire kidney graft. Perhaps despite the scores 2 and 3 in CI or CT in biopsy specimens, the function of the kidney as a whole is sufficient enough for a graft recipient. Another explanation is that the presence of the IF/TA changes does not indicate an active process of fibrinogenesis, which is rather evidenced by the activity of the studied EMMPRIN and possibly other markers of the ECM turnover. Additionally, CD147 levels correlated positively with creatinine concentrations and negatively with eGFR [23]. One experimental study on murine models established that mice deprived of the gene coding for CD147 had far fewer fibrotic lesions in the kidneys at 14 days after bilateral ureteral occlusion. Additionally, no expression of MMP-2, an element of key importance to EMT, was found in the models [24]. CD147 is a strong stimulator of MMP-2, an enzyme that in children persists at increased levels since the beginning of renal injury and during its progression correlates with the concentration of TGF-β, the main stimulator of fibrosis. MMP-2 is believed to be an indicator of exacerbated cellular damage, inflammation, and elevated proteolytic processes in children with CKD [25]. The positive correlation between EMMPRIN levels and MM lesions in the glomeruli that were found in our study reflected the effect of CD147 on increased ECM amounts in the glomerular spaces between the mesangial cells. This effect may be exerted by a decreased activity of metalloproteinases. Other histopathological lesions that CD147 correlates with, namely CG and AH, do not lend themselves to easy interpretation. CG may be present in the picture of many pathologies, such as chronic or chronic active humoral rejection, thrombotic microangiopathy, hypertension-related glomerulopathy, and glomerulonephritis. According to BANFF classification criteria from 1997, as updated, AH is a lesion of “uncertain significance” [26]. The positive correlation between CD147 levels and urine protein concentrations is an important aspect of our results. Proteinuria is a known marker for kidney diseases. Anti-proteinuria treatment improves the prognosis for renal function. However, apart from being a marker, proteinuria is also a factor contributing to the progression of renal failure [27,28,29]. By exacerbating kidney injury, CD147 increases proteinuria, which has an additional exacerbating effect on the progression of renal allograft failure. Similar results were obtained by Yoshika et al., who established a positive correlation between urine CD147 levels and urine protein concentrations [30].

### 4.5. Future Perspectives

Since biopsy performance is invasive and not commonly practiced and traditional kidney disease markers (i.e., eGFR and albuminuria) are not fully suitable for early detection of kidney injury and prediction of developing progressive kidney disease, there is an urgent need for the identification of novel, non-invasive biomarkers that could easily detect patients at risk for declined kidney graft function. Disrupted kidney ECM turnover has been identified as a contributing factor to increased fibrogenesis. Therefore, ECM proteins could serve as adequate biomarkers. In future studies, we propose further studies of matrix-metalloproteinases (MMPs), and their counteractors—the tissue-inhibitors of metalloproteinases (TIMPs) alongside EMMPRIN. For example, MMP-9 and MMP-2 have been elevated in various kidney disease models and humans, and urinary TIMP-1 has already been proposed as a marker of kidney function in patients after kidney transplantation and also its expressions have been increased in patients with CKD [31,32,33,34]. TGFβ is another well-known profibrotic factor, and in available research, its urinary concentration correlated positively with fibrotic changes in kidneys. Interestingly, studies regarding the metalloproteinases and their inhibitors have concluded that MMP-2 is responsible for TGFβ activation. Furthermore, TGFβ stimulates the production of MMP-2 by activating membrane-type 1 matrix metalloproteinase (MT1-MMP/MMP-14) [35]. Other markers that could be evaluated are monocyte chemoattractant protein 1 (MCP-1), Kidney Injury Molecule-1 (KIM-1), and neutrophil gelatinase-associated lipocalin (NGAL) [31,35]. We strongly believe that creating a panel of biomarkers of ECM turnover could be sufficient for accurately identifying patients at risk of progressive graft failure.

## 5. Conclusions

In conclusion, EMMPRIN/CD147 displays adverse effects on renal graft survival. This study reports that higher serum EMMPRIN/CD147 concentrations are associated with the more frequent occurrence of DGF, poorer short-term (1- and 2-years post-Tx) and long-term (69 months post-Tx on average) renal graft function, more profound IF/TA lesions in biopsy specimens, and the degree of proteinuria. Hence, authors postulate that EMMPRIN/CD147 could serve as an easily available, non-invasive marker for predicting the course of the post-Tx period, especially in terms of renal graft function. Authors postulate the novelty of the research as there has only been one article previously published regarding the role of EMMPRIN and IF/TA changes in kidney grafts. However, authors are aware that larger studies need to be conducted to introduce the EMMPRIN/CD147 to clinical practice.

## 6. Limitations

There are some limitations regarding our study. Firstly, the retrospective character of the study and small sample size should be acknowledged. Secondly, the different times between Tx, performing a kidney biopsy, and data collection may lead to a minor bias in data acquisition and results. Thirdly, for our study, we estimated the GFR using the MDRD formula, and neither the CKD-EPI formula currently recommended by the 2012 KDIGO guidelines nor the cystatin c clearance formula were used. We also faced some obstacles to discussing our results in comparison to others, as the data concerning the impact of EMMPRIN on kidney graft function are limited.

## Figures and Tables

**Table 1:** The thresholds for evaluated Banff Lesions Score according to Banff Classification 2018.

Banff Lesion	Abbreviation	0	1	2	3
Interstitial inflammation	I	<10%	10–25%	26–50%	>50%
Tubulitis	T	none	1–4/tubular cross section or 10 tubular epithelial cells	5–10	>10 or foci of tubular basement membrane destruction with i ≥ 2 and t2 elsewhere
Glomerulitis	G	none	<25%	25–75%	>75%
Peritubular capillaritis	PTC	<3 leukocytes/PTC	≥1 leukocyte in ≥10% of PTCs with max. of 3–4/PTCS	≥1 leukocyte in ≥10% of PTCs with max. of 5–10/PTCS	≥1 leukocyte in ≥10% of PTCs with max. of >10/PTCS
C4d	C4d	none	<10%	10–50%	>50%
Interstitial fibrosis	CI	≤5%	6–25%	26–50%	>50%
Tubular atrophy	CT	none	≤25%	26–50%	>50%
Vascular fibrous Intimal thickening	CV	none	≤25%	26–50%	>50%
double contours of GBM	CG	none	1 a: only by EM 1 b: ≤25% by LM	26–50%	>50%
Mesangial matrix expansion	MM	none	≤25%	26–50%	>50%
Arteriolar hyalinosis	AA	none	Mild to moderate ≥1	Moderate to severe in >1	Severe in many
Hyaline arteriolar thickening	AAH	none	1 without circumferential	≥1 without circumferential	Severe in many circumferenial

**Table 2:** Clinical characteristics of the study group.

	N	ME	MIN	MAX	MEAN	SD
Time from Tx (months)	49	69	12	182	75.7	52.3
Recipient age (years)	49	42	24	71	45.7	13.6
BMI at Tx	49	23.8	16.1	34.8	24.4	4
BMI at last appointment	49	25.4	18.1	34	25.9	4.3
eGFR at last appointment (mL/min/1.73 m^2^)	49	36	15	89	38.6	19.1
Urine protein (mg/dL)	49	0	0	865.7	78.7	165
Duration of dialysis prior to Tx (months)	49	15	0	102	23.9	23.8
CIT (min)	49	1260	72	2100	1116.7	555
PRA(%)	49	3	0	56	8	15.9
ZENITH eGFR (mL/min/1.73 m^2^)	49	31.0	98.0	118.0	58	17
eGFR at 1 year (mL/min/1.73 m^2^)	49	45	9	96.0	47.7	17.9
eGFR at 2 years (mL/min/1.73 m^2^)	48	49	15	85	48	17.1
eGFR at 3 years (mL/min/1.73 m^2^)	43	47	17	87	47.3	15.4
eGFR at 4 years (mL/min/1.73 m^2^)	38	46	19	98	48	18.9
eGFR at 5 years (mL/min/1.73 m^2^)	35	41	17	109	45.5	20.1
eGFR at 10 years (mL/min/1.73 m^2^)	13	44	23	82	47.8	18.7
EMMPRIN (pg/mL)	49	6623.2	643.36	59,861.6	9419.67	9678.712
sex	49 (23 W,26 M)					

N—sample size; ME—median; MIN—minimum; MAX—maximum; SD—standard deviation; Tx—renal transplantation; BMI—body mass index; eGFR—estimated glomerular filtration rate (estimated using the MDRD formula); ZENITH eGFR—the highest estimated glomerular filtration rate within the first 6 months post-renal transplantation (estimated using the MDRD formula); W—female group; M—male group.

**Table 3:** Characteristics of the groups: with and without chronic changes in allograft biopsy specimens.

Group without Chronic Changes N = 23				Group with Chronic Changes N = 26			
	ME	MIN	MAX	ME	MIN	MAX	p
Time from Tx (months)	44.9	3.16	162.1	82.7	9.53	181	0.004
Recipient age (years)	43	24	71	40,5	26	70	NS
BMI at last appointment (kg/m^2^)	26.2	18.7	33.7	24.4	18.1	34	NS
eGFR at last appointment (mL/min/1.73 m^2^)	41.9	6.1	88.9	33.7	6.02	71.62	NS
ZENITH eGFR (mL/min/1.73 m^2^)	64	34.17	92.38	53.85	31.1	97.76	0.03
G	0	0	2	0	0	0	NS
CG	0	0	3	0	0	3	NS
MM	0	0	3	0	0	3	0.013
I	0	0	3	1	0	3	0.007
T	0	0	2	0	0	3	NS
PTC	0	0	10	0	0	3	NS
CV	0	0	3	0	0	2	NS
AH	1	0	3	1	0	3	0.027
CI	0	0	1	2	0	3	3.32 × 10^−6^
CT	1	0	1	2	0	3	4.06 × 10^−8^

N—sample size; ME—median; MIN—minimum; MAX—maximum; Tx—renal transplantation; BMI—body mass index; eGFR—estimated glomerular filtration rate (estimated using the MDRD formula); ZENITH eGFR—the highest estimated glomerular filtration rate within the first 6 months post-renal transplantation (estimated using the MDRD formula); G—glomerulitis; CG—double contours of GBM; MM—mesangial matrix expansion; I—interstitial inflammation; T—tubulitis; PTC—peritubular capillaritis; CV—vascular fibrous intimal thickening; AH—arterioral hyalinosis; CI—interstital fibrosis; CT—tubular atrophy; *p*—*p* value; NS—not statistically significant.

**Table 4:** EMMPRIN values in in DGF POSITIVE and DGF NEGATIVE group.

	DGF Positive					DGF Negative					
	ME	MIN	MAX	MEAN	SD	ME	MIN	MAX	MEAN	SD	*p*
EMMPRIN(pg/mL)	7729.9	643.4	29,709.47	11,658.36	8370.301	5348.1	897.4	59,861.6	8047.789	10,064.92	0.00896
N	16					33					

N—sample size; DGF—delayed graft function; min—minimum; max—maximum; ME—median; SD—standard deviation; *p*—*p* value.

## Data Availability

Not available.
